# Artificial dielectric polarizing-beamsplitter and isolator for the terahertz region

**DOI:** 10.1038/s41598-017-06297-7

**Published:** 2017-07-19

**Authors:** Rajind Mendis, Masaya Nagai, Wei Zhang, Daniel M. Mittleman

**Affiliations:** 1 Brown University, School of Engineering, Providence, RI 02912 USA; 2 Osaka University, Graduate School of Engineering Science, Toyonaka Osaka, 560-8531 Japan

## Abstract

We demonstrate a simple and effective strategy for implementing a polarizing beamsplitter for the terahertz spectral region, based on an artificial dielectric medium that is scalable to a range of desired frequencies. The artificial dielectric medium consists of a uniformly spaced stack of metal plates, which is electromagnetically equivalent to a stacked array of parallel-plate waveguides. The operation of the device relies on both the lowest-order, transverse-electric and transverse-magnetic modes of the parallel-plate waveguide. This is in contrast to previous work that relied solely on the transverse-electric mode. The fabricated polarizing beamsplitter exhibits extinction ratios as high as 42 dB along with insertion losses as low as 0.18 dB. Building on the same idea, we also demonstrate an isolator with non-reciprocal transmission, providing high isolation and low insertion loss at a select design frequency. The performance of our isolator far exceeds that of other experimentally demonstrated terahertz isolators, and indeed, even rivals that of commercially available isolators for optical wavelengths. Because these waveguide-based artificial dielectrics are low loss, inexpensive, and easy to fabricate, this approach offers a promising new route for polarization control of free-space terahertz beams.

## Introduction

A polarizing beamsplitter (PBS) is a device that splits an arbitrarily polarized optical beam into two orthogonal, linearly polarized components. In the terahertz (THz) spectral region, there have been only a few studies on PBSs, using metamaterials^1^, dielectric bi-layers^2^, diffraction gratings^3^, and recently, using form birefringence^4^. In all of these cases, the fabrication of the device is complicated and not readily scalable. Here we present experimental characterization of a PBS involving a far simpler geometry and exhibiting remarkable performance. Our design is based on artificial dielectrics, man-made media that mimic properties of naturally occurring dielectric media, or even manifest properties that cannot generally occur in nature. Although originally, briefly introduced by the microwave community^5–7^, the wavelength scaling that result when transitioning from microwaves to THz waves gives new life to this waveguide-based technology^8^.

At the design frequency of 0.2 THz, our PBS exhibits an extinction ratio of 42 dB in transmission and 28 dB in reflection with an overall insertion loss of 0.18 dB. These values rival the specifications of polarizing cube beamsplitters that are commercially available for visible and near-infrared wavelengths. Furthermore, by combining our PBS with a quarter-wave plate based on the same artificial-dielectric technology, we demonstrate a THz isolator with an isolation of 52 dB and an insertion loss less than one dB, at a frequency of 0.46 THz. This isolation is more than three orders of magnitude higher than recently demonstrated THz isolators based on graphene^9^, and the insertion loss is considerably lower than previously demonstrated THz isolators based on ferrite materials^10, 11^. In addition, our design does not require an externally applied magnetic field. Indeed, the performance of our device rivals that of commercially available Faraday isolators for optical wavelengths. This simple method for achieving very high isolation will be invaluable for numerous applications involving high-power THz sources^12^ or THz systems with highly sensitive receivers^13^.

## Design and Fabrication

**Figure 1:**
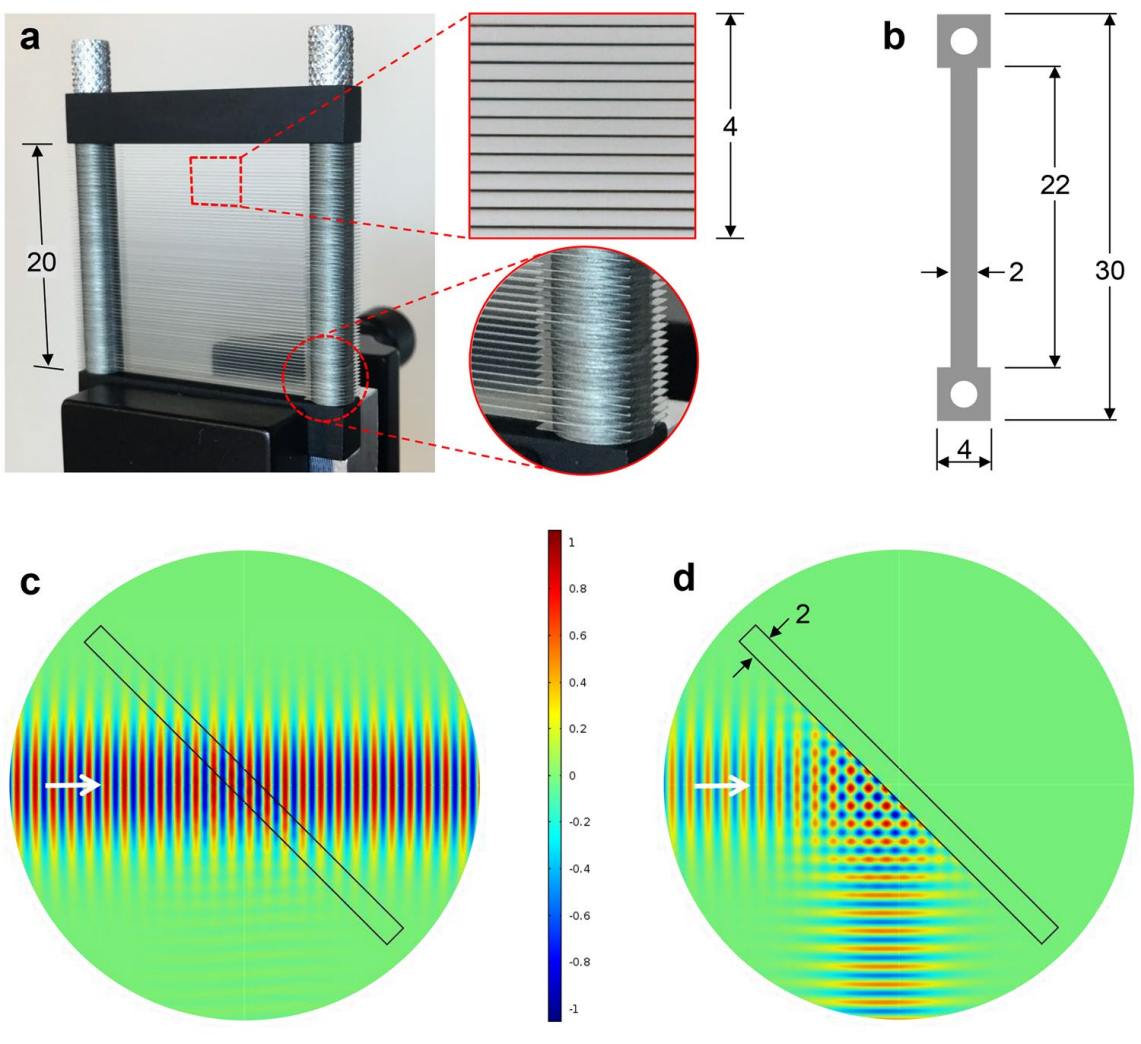
Fabricated device and simulations. (**a**) Photograph of the PBS device with close-up views showing a square section of the stack-of-plates (looking on axis) and the bottom-corner of the mounting post. (**b**) Geometry of a single stainless-steel plate. (**c**,**d**) FEM simulations of the beam propagation at a frequency of 0.2 THz when the input electric field is linearly polarized (**c**) perpendicular, and (**d**) parallel, to the plates. The beam diameter is 1 cm and the angle of incidence is 45°. All dimensions are in millimeters.

The artificial dielectric medium consists of a uniformly spaced stack of identical, rectangular metal plates. This stack-of-plates is electromagnetically equivalent to a stacked array of parallel-plate waveguides (PPWGs)^8^. The plates are made of 30 µm thick stainless steel and are spaced 300 µm apart, as seen in the prototype device shown in Fig. 1(a). This aspect ratio of 1:10 between the plate thickness and the plate spacing was chosen to maximize device efficiency while at the same time, achieving mechanical robustness. Unnecessarily thick plates would result in undesirable reflection losses and overly thin plates may not provide adequate robustness to realize the required uniform plate spacing. The plates and the spacers are fabricated by chemical etching to avoid any strain or burring, which helps to maintain their flatness. The device is assembled by stacking the plates and spacers alternating along two locating posts, such that the plates are free-standing, supported only by their ends. At each end, there is an integrated square pad with a mounting hole as shown in Fig. 1(b), which illustrates the actual shape of a plate. Once assembled, this stacked-plate arrangement results in a clear aperture of 20 mm. The magnified, close-up view of the 4 mm square section of the clear aperture illustrates the flatness of the plates and the uniformity of their spacing. Taking a slice across this image and using a graphical reconstruction method, we measure an average center-to-center plate spacing of 330 μm, with a standard deviation of 5 μm.

In the designed PBS device geometry, the THz beam is directed at the stack-of-plates at an angle of 45° to the virtual surface emulated by the plate edges, with the plane of incidence parallel to the plate surfaces. The operation of the PBS relies on both the TEM and TE_1_ fundamental modes of the PPWG^14, 15^. When the input electric-field is linearly polarized perpendicular to the plates (*s*-polarized), only TEM modes are excited in the PPWG array, and the beam propagates through the device without altering its path. This behavior is illustrated in the COMSOL FEM simulation result shown in Fig. 1(c), which plots the instantaneous electric field of the propagating beam along the axial cross-section parallel to the plate surfaces, at a frequency of 0.2 THz. As long as the input beam diameter is sufficiently larger than the plate spacing (for proper mode-matching) and the interaction path-length is short, this TEM-mode propagation will be a very efficient (i.e., low loss) process^14^. On the other hand, when the input electric field is linearly polarized parallel to the plates (*p*-polarized), only TE_1_ modes can be excited in the PPWG array, and the propagation is governed by the mode’s cutoff frequency. Input frequencies that are above the cutoff will propagate through the device, while those that are below the cutoff will be reflected. In fact, these below-cutoff frequencies will be *totally* and *specularly* reflected in a well-defined beam^8^. This behavior is illustrated in the FEM simulation result shown in Fig. 1(d), which plots the instantaneous magnetic field of the propagating beam at a frequency of 0.2 THz.

**Figure 2:**
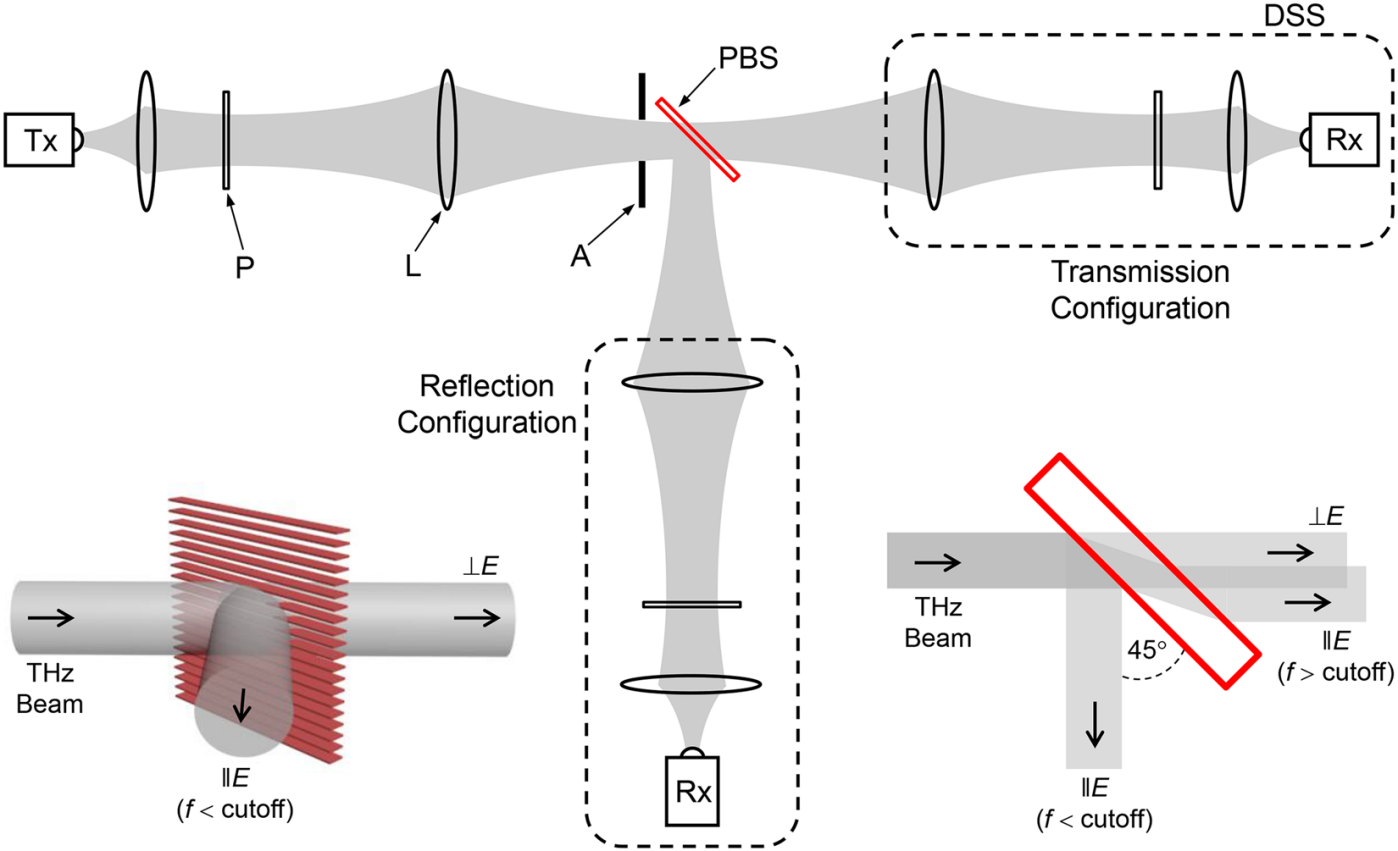
Schematic of the experimental setup. The plane of the paper corresponds to the horizontal plane, which is also the plane of the plates. Gray areas represent the propagating beam. Here, L: lens, A: aperture, P: polarizer, Rx: receiver, Tx: transmitter, DSS: detector subsystem, and PBS: polarizing beamsplitter. The complete DSS (shown within the black dashed enclosure) can be moved intact, from the on-axis position to the 90°-off-axis position to measure either transmission or reflection. All the polyethylene lenses are in confocal configurations to achieve maximum power transfer through the optical system. The right inset diagram illustrates the general propagation behavior of the device (not drawn to scale and exaggerated for clarity) for an input beam with an arbitrary linear polarization direction and a broad spectrum having frequencies extending above and below the TE_1_-mode’s cutoff. The left inset is a 3D rendering of the device geometry showing the stack-of-plates [in relation to Fig. 1(a)] when operated in the PBS configuration.

Under oblique incidence, the TE_1_-mode cutoff frequency is given by *c*/(2*b* cos *α*), where *c* is the free-space velocity, *b* is the plate spacing, and *α* is the incidence angle^16^. For the demonstrated device, the cutoff is at 0.7 THz when the device is illuminated at 45°. Now, if the input electric-field is linearly polarized at an arbitrary angle (between 0° and 90°) to the plates, both the TEM and TE_1_ modes are excited simultaneously. Then, the portion of the input beam (the perpendicular component) propagating via the TEM mode exits the device on axis, polarized perpendicular to the plates. This TEM-mode contribution is independent of the frequency. In contrast, the portion that could excite the TE_1_ mode (the parallel component), if below cutoff, is totally reflected at 90° to the input axis, polarized parallel to the plates. By varying the angle of the input polarization, we can control the power division into the two output arms, thereby realizing a versatile PBS. Incidentally, if there is any parallel component at a frequency above the cutoff, this portion would propagate through the device via the TE_1_ mode and exit the device with a slight lateral shift, polarized parallel to the plates. The lateral shift is caused by the refraction of the beam inside the device due to the lower refractive index compared to free-space^16^. This general behavior of the device for an incident beam with an arbitrary linear polarization direction having frequencies extending below and above the cutoff is schematically illustrated in the right inset of Fig. 2. Since the PBS operation would be hampered by any excitation of the TE_1_ mode, the upper limit of the operational bandwidth of the PBS is set by the mode’s cutoff frequency. Therefore, it follows that the operational bandwidth can be increased by decreasing the plate spacing and/or increasing the incidence angle.

## Experimental Characterization - PBS

The prototype PBS device was experimentally investigated in both transmission and reflection configurations using a THz time-domain spectroscopy system, as schematically depicted in Fig. 2. In this spectroscopy system, both the transmitter and receiver modules are fiber coupled to the main controller unit, so as to accommodate the multiple polarization axes and spatial configurations. Throughout the experiment, the device was located between two Norcada wire-grid polarizers to purify the input and detected linear polarizations. Via external optics, the input beam was formed to a frequency-independent 1/*e*-amplitude Gaussian diameter of approximately 10 mm and was made to enter the device fairly well collimated. The same optical arrangement was employed for the output beam to maintain input-output symmetry. While the input optics were fixed in space, the detector sub-system could be moved (intact) from the on-axis position to the 90°-off-axis position to change from a transmission configuration to a reflection configuration. A 16 mm diameter aperture was situated in close proximity to the input transverse-plane of the device. This eliminated any energy “spill-over”, providing a true reference signal when the device was not in the beam path, and also served as a marker for the beam axis. In addition to three-axis linear translation, the device mount also included a precision rotation stage to adjust the incidence angle in the horizontal plane, along with precision control of the tilt in two perpendicular vertical planes, allowing complete three-axis rotational positioning.

**Figure 3:**
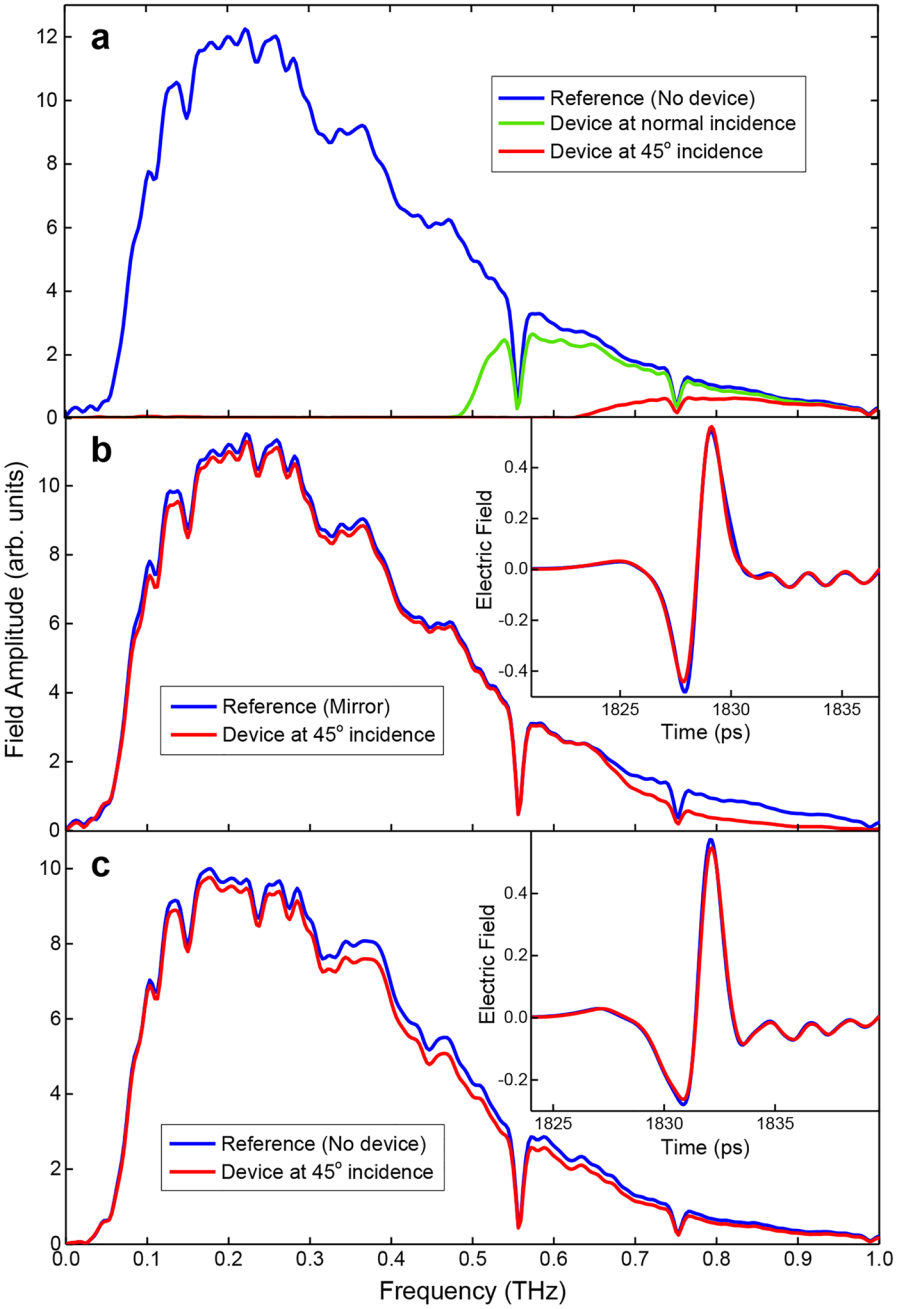
Transmission and reflection spectra. (**a**) Transmission spectra when the input polarization is parallel to the plates. The blue curve is the reference with no device, the green curve is with the device at normal incidence, and the red curve is with the device at 45° incidence. The device spectra correspond to TE_1_-mode propagation through the device. The sharp dips at 0.56 THz and 0.75 THz are due to water-vapor absorption. (**b**) Reflection spectra when the input polarization is parallel to the plates. The red curve is with the device at 45° incidence and the blue curve is the reference with a polished aluminum mirror replacing the device. (**c**) Transmission spectra when the input polarization is perpendicular to the plates. The blue curve is the reference with no device and the red curve is with the device at 45° incidence. The device spectrum corresponds to TEM-mode propagation through the device. The insets in (**b**) and (**c**) show the corresponding time-domain signals.

Figure 3 illustrates various measured amplitude spectra that were obtained by Fourier transforming the detected time-domain signals. Figure 3(a) shows spectra corresponding to the purely TE_1_-mode behavior of the device in transmission. During this measurement, both the transmitter and receiver polarization axes (along with the input and output polarizer axes) were kept horizontal. Here, the blue curve corresponds to the reference signal when there is no device in the path of the beam. The sharp dips seen at 0.56 THz and 0.75 THz are due to water vapor absorption. The green curve corresponds to the signal when the device is in the path of the beam at normal incidence. This spectrum indicates a cutoff near 0.5 THz, as expected for a 300 μm plate spacing. The red curve corresponds to the signal when the device is at 45° incidence (the designed operating configuration), and as predicted by theory, the cutoff shifts to a value near 0.7 THz. This TE_1_-mode diagnostic measurement is indicative of the quality of the device and serves to estimate the operational bandwidth of the PBS.

For the spectra in Fig. 3(b), the polarization axes of the transmitter, receiver, and the polarizers were maintained horizontal as before, but the detector sub-system was moved to measure the reflected signal. The red curve corresponds to the reflected signal when the device is at 45° incidence. The blue curve corresponds to the reference signal when the device is replaced by a polished aluminum mirror. The inset gives the detected time-domain signals. Along with the spectra, they prove the highly efficient and non-dispersive broad-band operation of the device in reflection. The high-frequency attenuation of the device spectrum which appears to build up starting close to 0.7 THz is consistent with the TE_1_-mode transmission spectrum in Fig. 3(a). Since this attenuation manifests for relatively low amplitude levels of the input spectrum (as evident by the reference), there is only minimal change in the reflected temporal signal.

For the spectra in Fig. 3(c), the measurement configuration was changed back to transmission, and the polarization axes of the transmitter, receiver, and the polarizers, were rotated to be vertical. Therefore, this configuration investigates the purely TEM-mode behavior of the device. The blue curve corresponds to the reference signal when there is no device in the beam path. The red curve corresponds to the signal when the device is at 45° incidence to the input beam. The detected time-domain signals are given in the inset, and as before, along with the amplitude spectra, prove the highly efficient and non-dispersive broad-band operation of the device in transmission. This observation is not surprising since the TEM mode of the PPWG is a very low-loss and dispersion-less propagating mode^14^. However, it should be noted that in order to obtain this efficient propagation it was important for the collimated beam axis to be aligned so as to be parallel to the plate surfaces with high accuracy, and also for the input polarization direction to be exceptionally well perpendicular to the plate surfaces. Deviations from these two conditions (by more than a few degrees) would result in additional losses, as the oblique incidence results in a longer interaction path-length, compared to that with normal incidence. It is not only the added ohmic loss that comes into play here, but also the relative parallelism of the stack of plates.

**Figure 4:**
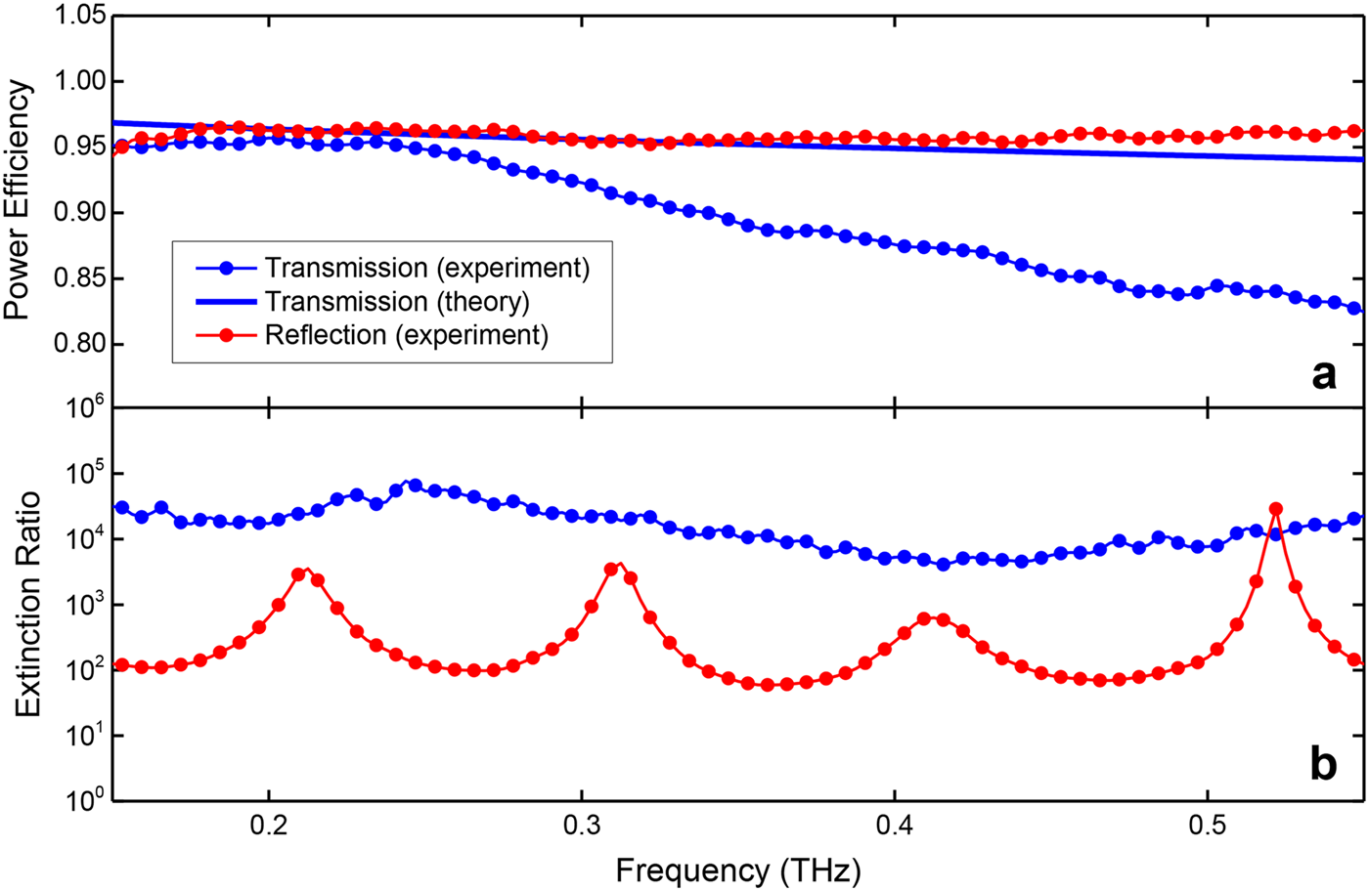
Power efficiency and extinction ratio. (**a**) Power efficiency for the transmission (blue dots) and reflection (red dots) arms, within the operational bandwidth. The blue solid curve gives the theoretical efficiency for the transmission arm taking into account only the ohmic loss. (**b**) Cross-polarization extinction ratio for the transmission (blue dots) and reflection (red dots) arms, within the operational bandwidth.

Using the spectra in Figs. 3(b) and (c), we can deduce the power efficiency of the device for the transmission and reflection arms. These efficiency curves are plotted in Fig. 4(a) by the blue and red dots for transmission and reflection, respectively, within the operational bandwidth of the PBS. For the reflection arm, the efficiency curve is relatively flat throughout the bandwidth, and indicates a power efficiency of 96% at both 0.2 THz and 0.5 THz, for example. This corresponds to an insertion loss of only 0.18 dB. For the transmission arm, the efficiency is 96% at 0.2 THz, and drops to 84% at 0.5 THz. This corresponds to an insertion loss of 0.76 dB. For comparison, also plotted is the theoretical transmission (blue solid curve) taking into account only the ohmic loss associated with TEM-mode propagation. The discrepancy with the experimental curve (especially as the frequency increases) implies that there are other sources of loss. Part of this extra loss is caused by the non-negligible impedance mismatch at the input and output surfaces of the device, even in the case of TEM-mode propagation^17^. This gives rise to two small reflections from these virtual surfaces, which may also be affected by the finite thickness of the plates. In fact, these reflections played a role in the subsequent measurements that were carried out to estimate the cross-polarization extinction ratios of the PBS.

In the next measurement, the input polarization was oriented at 45° to the horizontal plate surfaces, and both the vertical and horizontal components of the output were measured, for both the transmission and reflection configurations separately. Therefore, for the transmission arm, in addition to the major component of the output that is polarized perpendicular to the plates, this also measures the minor component that is polarized parallel to the plates. This minor cross-polarization component is a result of energy leakage due to subtle device imperfections. The squared ratio of these two components gives the extinction ratio, which is plotted by the blue dots in Fig. 4(b). This curve indicates extinction ratios of 42 dB and 39 dB at 0.2 THz and 0.5 THz, respectively. Similarly, for the reflection arm, in addition to the major component polarized parallel to the plates, this measures the minor component polarized perpendicular to the plates. In this case, the cross-polarization component is due to the two TEM-mode reflections at the input and output surfaces, as discussed above. The estimated extinction ratio is plotted by the red dots in Fig. 4(b), where the observed ripple is due to the associated Fabry-Perot effect. This curve indicates extinction ratios of 28 dB and 22 dB at 0.2 THz and 0.5 THz, respectively. These values are not as impressive as for the transmission arm; however, a simple way to improve this extinction would be to add a polarizer to the reflection arm. This polarizer could be an identical artificial-dielectric device as used for the PBS, and would be extremely efficient since the beam would now be at normal incidence.

**Figure 5:**
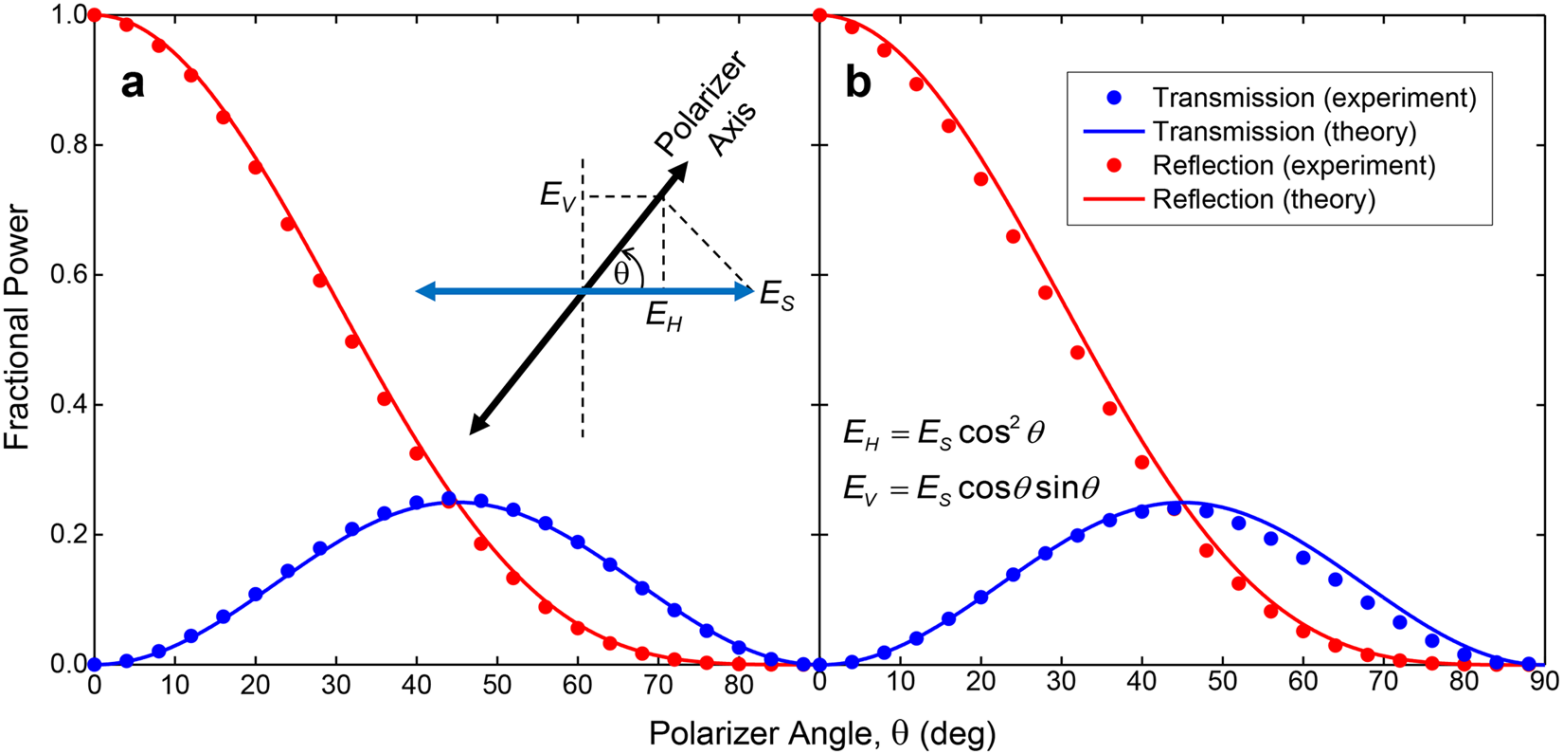
Fractional power division. Results are shown for the frequencies of (**a**) 0.2 THz, and (**b**) 0.5 THz, as a function of the input-polarizer angle. The dots give the experimental values and the solid curves give the theoretical values. The red curves correspond to the reflection arm (horizontal polarization) and the blue curves correspond to the transmission arm (vertical polarization). The inset cartoon shows the respective directions of the input-side polarizer axis and the transmitter electric-field (*E*_S_) polarization that is fixed, and the consequent horizontal (*E*_H_) and vertical (*E*_V_) components of the electric field as a function of the polarizer angle.

The final characterization step of the PBS was to measure the power division into the two output arms as a function of the input polarization angle. Here, the input polarization is initially set parallel to the plate surfaces by the transmitter, and is rotated by the input-side polarizer in steps of 4°. Then, in the reflection configuration, the output is detected with the output-side polarizer (and receiver) oriented parallel to the plates. Similarly, in the transmission configuration, the output is detected with the output-side polarizer (and receiver) oriented perpendicular to the plates. The experimental results are given by the dots in Fig. 5(a) and (b) for the frequencies of 0.2 THz and 0.5 THz, respectively. These agree very well with the theoretical power dependences of cos^4^ *θ* and cos^2^ *θ* sin^2^ *θ* given by the solid curves for the reflection and transmission arms, respectively. These results confirm that we can achieve any power division simply by rotating the input polarization axis, hence, a versatile PBS.

## Experimental Characterization - Isolator

**Figure 6:**
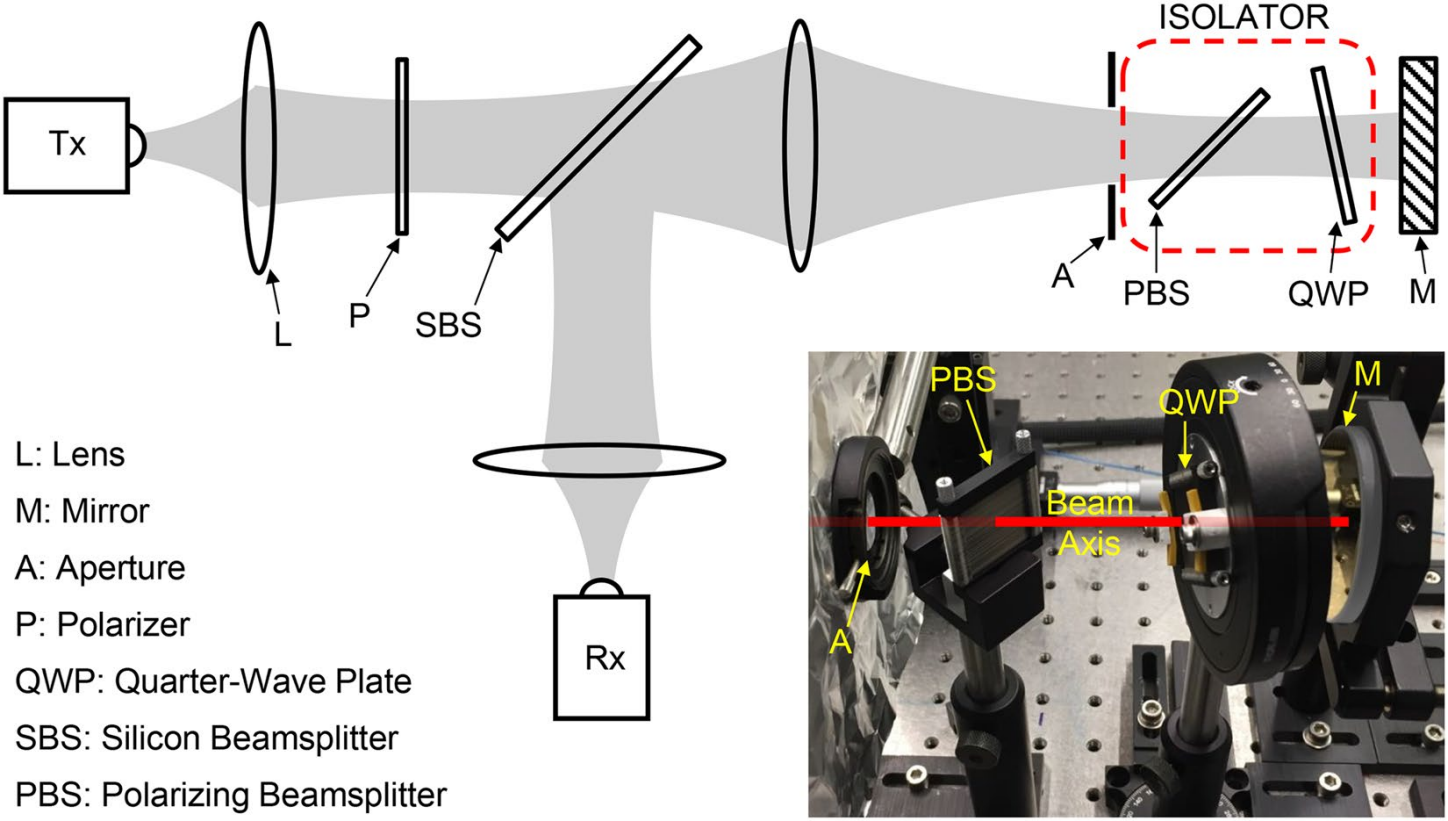
Schematic of the experimental setup used to investigate the isolator. The isolator consists of the PBS combined with a QWP as shown by the red dashed enclosure. A photographic view of the isolator is shown inset. The input polarization is vertical (and perpendicular to the plane of the paper). A gold mirror was used to create the back-reflection, and a silicon beamsplitter was used to tap it off to the receiver. The PBS is at 45° incidence by design, and the QWP is at a 12° incidence to eliminate reflections originating from it.

In order to demonstrate an advanced device application of the PBS, we constructed an isolator for the THz region. In optics, the primary purpose of an isolator is to minimize or eliminate feedback (back-reflections), while transmitting sufficient power in the forward direction. Isolators are essential for the stable and reliable operation of lasers, especially high-power ones, in well-aligned complex optical systems, where back-reflections are inevitable. High-contrast isolators are also critical components in full-duplex communication systems^18^. In the THz region, there have been only a few experimental studies on isolators, using ferrite materials^10, 11^, and graphene^9^. Our THz isolator is designed by combining the original PBS with a quarter-wave plate (QWP) that is also fabricated utilizing the same artificial-dielectric technology. This isolator design, a configuration commonly employed in optics^19, 20^, is shown by the red-dashed enclosure in the schematic diagram given in Fig. 6, which illustrates the experimental setup used to investigate its behavior.

The QWP was fabricated using the same 30 μm thick stainless-steel plates as before, but with a plate spacing of 1 mm in one design (QWP_1_), and a spacing of 0.8 mm in another design (QWP_2_). For the proper operation of the QWP, the plane of the plates is oriented at 45° to the input vertical polarization set by the PBS. Then, half of the input energy will propagate via the TEM mode and the other half via the TE_1_ mode. After propagating through the device at different velocities, these two orthogonal polarization components will acquire a relative phase of 90° at a certain frequency. It can be shown that this occurs at a frequency given by 0.5 *cd* [(1/*b*)^2^ + (0.5/*d*)^2^], where *d* is the propagation-path length. This is the isolation frequency of the isolator. Accordingly, since *d* = 2 mm, this should occur at 0.32 THz and 0.49 THz for QWP_1_ and QWP_2_, respectively. Now, when there is a back-reflection, due to the “double-pass” through the QWP, the phase difference will become 180°. This will effectively rotate the polarization axis of the resultant reflected beam by 90°^21^, rendering it horizontal, and be diverted in the off-axis direction by the PBS, essentially isolating it from the input-beam path.

**Figure 7:**
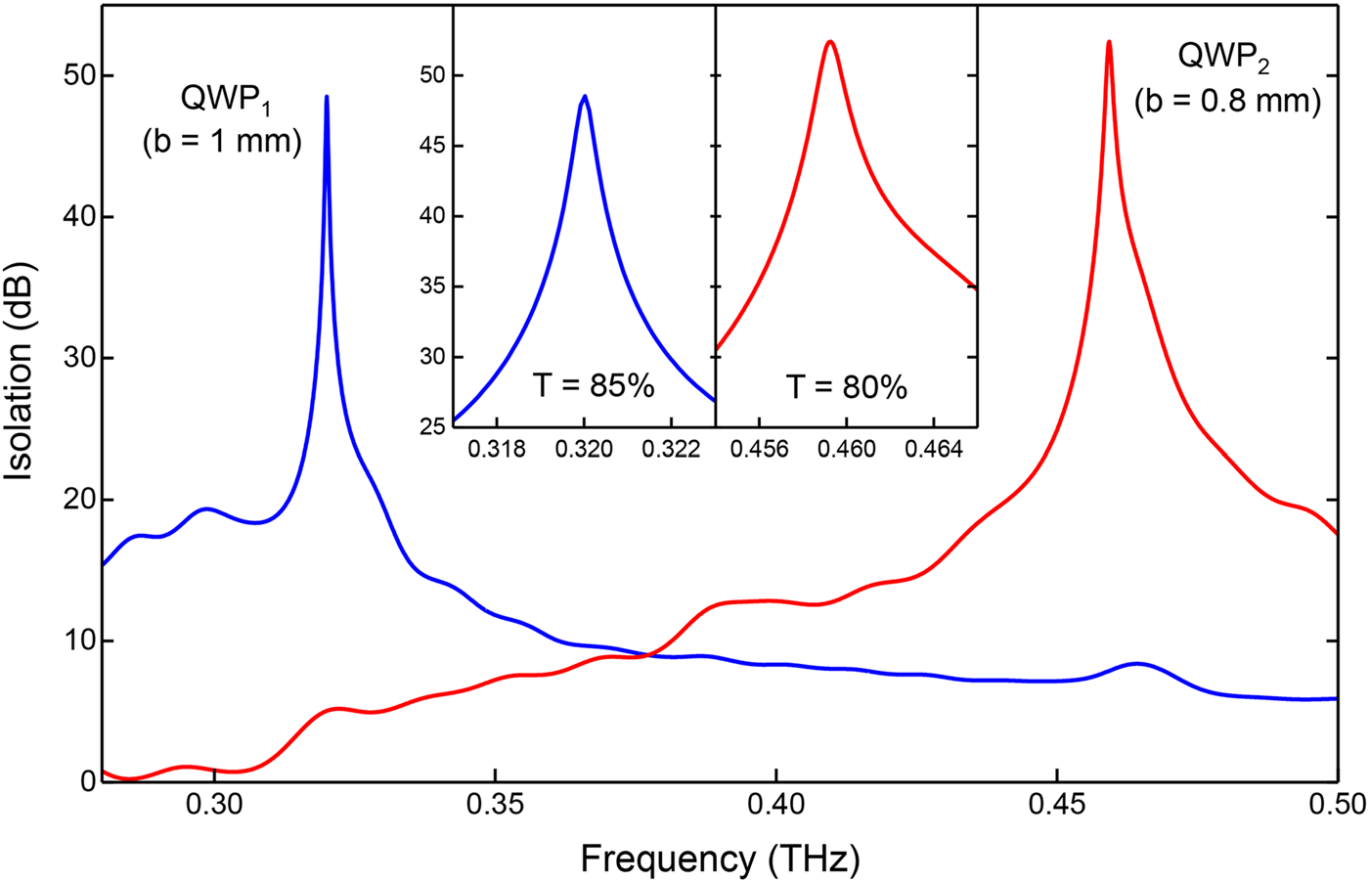
Measured isolation curves. Results are shown for the QWP_1_ (blue curve) and QWP_2_ (red curve) designs, with close-up views of the isolation peaks shown inset.

As shown in Fig. 6, a gold mirror was used to create the back reflection, and it was detected by tapping it off using a silicon beamsplitter, *with* and *without* the isolator in place. The ratio of these two spectra, after converting to decibels, gives the isolation curves, as plotted in Fig. 7. The QWP was mounted with an azimuthal tilt of 12° to eliminate the small reflections generated at its surfaces. (This tilt is seen in both the schematic and the photograph shown inset in Fig. 6.) For the QWP_1_ design, the maximum isolation is 48 dB, and this occurs at 0.32 THz, exactly as the theory predicts. For the QWP_2_ design, the maximum isolation is 52 dB, and this occurs at 0.46 THz, slightly shifted from the theoretical value. This discrepancy may be due to a weaker tightening of the plate assembly, resulting in a slightly larger than expected plate separation. For completeness, the forward power transmission of the isolator was also measured in a different transmission configuration, and it was 85% and 80% (equivalent to an insertion loss of 0.71 dB and 0.97 dB) for the QWP_1_ and QWP_2_ designs, respectively, at the peak isolation frequencies. A suitable arrangement that may provide continuous control of the plate spacing would allow dynamic tunability of the isolation-peak, adding versatility to the isolator.

## Summary

We have experimentally demonstrated a highly efficient and versatile PBS for the THz spectral region based on artificial dielectrics. The device geometry is exceedingly simple compared to all previous PBS attempts for this spectral region. The PBS exhibits insertion losses as low as 0.18 dB and cross-polarization extinction ratios as high as 42 dB. By combining this PBS with a QWP based on the same artificial-dielectric technology, we also demonstrate a THz isolator with peak isolations as high as 52 dB, rivaling the performance of commercial optical isolators. Furthermore, since the devices are made from stacked metallic plates, as opposed to dielectric materials, they also uniquely possess extremely high power handling capabilities, limited only by the breakdown of air within the plates. This would be valuable in applications where high THz fields are present, and would be inherently important for isolators since they are usually used in high-power applications.

Moreover, these artificial-dielectric devices are much more robust and inexpensive than wire-grid polarizers, which are often used in the THz region despite their high cost since only a few other options are available. The stack-of-plates design is extremely versatile and agile, since the device performance is determined largely by the plate spacing which can be easily reconfigured simply by using different spacers between the metal plates. It is even possible to engineer devices with non-uniform plate spacing, which could be valuable for producing or controlling spatial chirp or non-uniform polarization profiles. With this versatility and ease of reconfigurability, combined with the remarkable performance characteristics demonstrated here, our PBS design offers a promising new route for highly effective, efficient, tunable, and inexpensive polarimetric devices for the THz region.

### Data Availability Statement

The datasets generated during and/or analyzed during the current study are available from the corresponding author on reasonable request.
